# Host-Guest Carbon Dots for Enhanced Optical Properties and Beyond

**DOI:** 10.1038/srep12354

**Published:** 2015-07-21

**Authors:** Ya-Ping Sun, Ping Wang, Zhuomin Lu, Fan Yang, Mohammed J. Meziani, Gregory E. LeCroy, Yun Liu, Haijun Qian

**Affiliations:** 1 Department of Chemistry and Laboratory for Emerging Materials and Technology, Clemson University, Clemson, South Carolina 29634-0973, USA

## Abstract

Carbon dots, generally small carbon nanoparticles with various forms of surface passivation, have achieved the performance level of semiconductor quantum dots in the green spectral region, but their absorption and fluorescence in red/near-IR are relatively weaker. Conceptually similar to endofullerenes, host-guest carbon dots were designed and prepared with red/near-IR dyes encapsulated as guest in the carbon nanoparticle core. Beyond the desired enhancement in optical properties, the host-guest configuration may significantly broaden the field of carbon dots.

Carbon dots (also called carbon quantum dots in some literature reports despite the absence of classically defined quantum confinement) have emerged as a new class of photoactive nanomaterials^1^,^2^,^3^,^4^, with their fluorescence properties resembling those typically found in conventional semiconductor nanocrystals or quantum dots (QDs)^5^. The structure of a carbon dot is relatively simple, generally a small carbon nanoparticle with various forms of surface passivation, among which the more effective has been the chemical functionalization with organic or polymeric species (Fig. 1) for carbon dots of bright fluorescence emissions^4^. In the green over the spectral region covered by green fluorescent protein (GFP), for example, the performance of existing carbon dots in terms of fluorescence quantum yields in solution or the image brightness at the individual dot level on a substrate has been found to be competitive to that of the presently dominating CdSe/ZnS QDs^6^. According to available experimental results, carbon dots are nontoxic^2^,^3^,^4^,^7^, certainly without the toxicity concerns associated with the heavy metal-containing semiconductor QDs. Therefore, there has been a growing interest in potential applications of carbon dots for fluorescence bioimaging *in vitro* and *in vivo*^2^,^3^,^4^,^7^,^8^,^9^,^10^,^11^,^12^. However, despite the extensive effort in the relevant research community, the development of carbon dots of high fluorescence quantum yields in the biologically more significant near-IR spectral region has found only limited success. This, combined with the generally lower absorptivity of carbon nanoparticles in the red/near-IR, suggests that new strategies are necessary in order to use the carbon dots platform for fluorescence probes of the desired red/near-IR performance.

In the work reported here we “borrowed” the concept from the field of endofullerenes^13^ by considering the core carbon nanoparticle in a carbon dot as a “solid-state pool” (*versus* the cavity in a fullerene) to trap or encapsulate chromophoric species of strong red/near-IR absorption and emissions. This host-guest configuration takes advantage of the small carbon nanoparticle as host being optically largely transparent in the corresponding spectral regions, which has actually been identified above as a shortcoming of currently available carbon dots in their serving as red/near-IR probes. The resulting host-guest carbon dots, denoted as G@CDots (Fig. 1), exhibited the desired absorption and fluorescence properties, as designed and expected.

## Results and Discussion

The thermal carbonization of organic precursors has been a popular approach for the synthesis of carbon dots^2^,^3^,^4^, in which a portion of the precursor organic species is converted into carbon nanoparticles and the remaining serves the function of surface passivation agents. Among various thermal processing options is the use of microwave irradiation^14^,^15^,^16^,^17^,^18^,^19^,^20^,^21^, which in principle creates carbonized seeds for their preferential absorption of the subsequent microwave energy towards the formation of the targeted carbon dot structure of a carbon nanoparticle with organic species on the surface for passivation. The microwave processing was adopted in this work for the “one-pot” synthesis of the G@CDots, with G denoting the selected fluorescent dyes of cresyl violet (CV), nile blue (NB), and zinc phthalocyanine (ZnPc).

Experimentally for the synthesis of CV@CDots, CV (20 mg) in an ethanol solution was mixed well with oligomeric polyethylene glycol of molecular weight ~900 (PEG_900_, 2 g), followed by the removal of ethanol via purging with nitrogen gas. The resulting mixture was placed in a commercial microwave oven and irradiated at 300 W for 20 min. Then, water was added to the reaction mixture with sonication to obtain a dark colored aqueous solution. The solution was centrifuged at 20,000 *g*, from which only a negligible amount of precipitate was observed and discarded. The supernatant was dialyzed in a membrane tubing (cutoff molecular weight ~1,000) against fresh water to remove unreacted starting materials and other small molecular species, yielding CV@CDots in an aqueous solution. The same processing protocol was applied to the preparation of NB@CDots and ZnPc@CDots, except that for the latter a 1:1 mixture of PEG_900_ and oligomeric polypropionylethyleneimine instead of neat PEG_900_ was in the mixture with ZnPc for microwave irradiation. The sample solutions were used for optical spectroscopy measurements and microscopy characterization.

**Figure 2:**
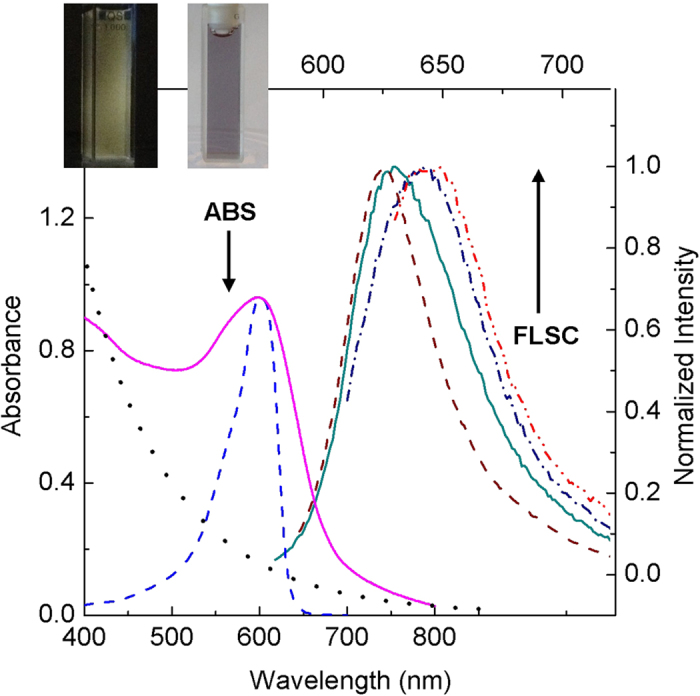
The absorption (ABS) spectrum of CV@CDots (—) and corresponding fluorescence (FLSC) spectra (excitation at 570 nm: —, 600 nm: -.-, and 620 nm: -..-) in aqueous solution. The spectra of free CV (- - -) and carbon dots from the carbonization of PEG_900_ without any encapsulation (…) in aqueous solutions are also shown for comparison. Inset: Photographs of an aqueous solution of the sample under UV light in the dark (left) and under natural day light (right).

**Figure 3:**
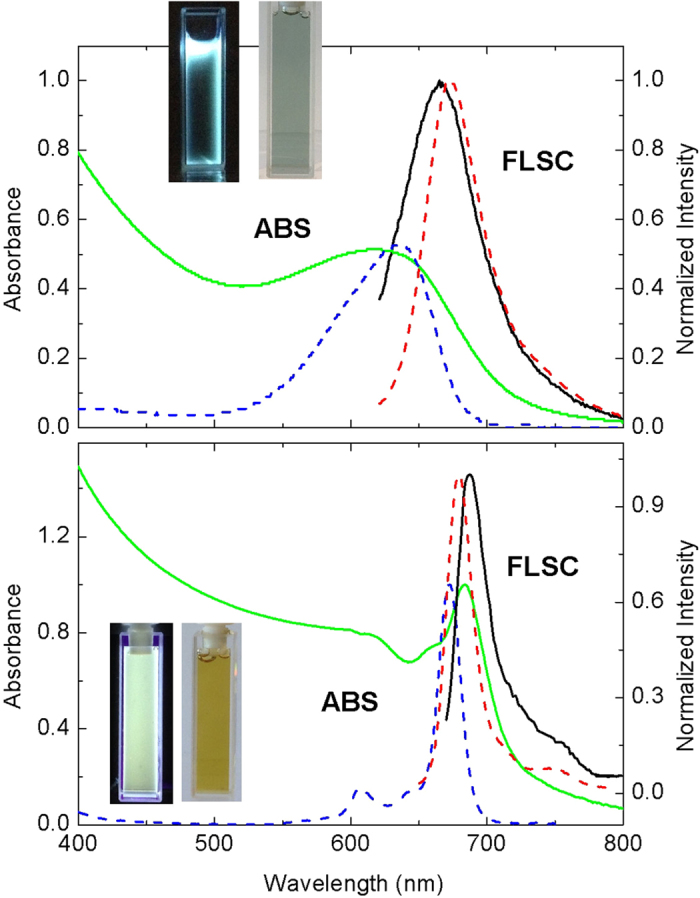
Absorption (ABS) and fluorescence (FLSC) spectra of NB@CDots (top, —) and ZnPc@CDots (bottom, —) and the corresponding free dyes (- - -) in aqueous solutions (except for free ZnPc in DMSO). Insets: Photographs of aqueous solutions of the corresponding samples under UV in the dark (left) and under natural day light (right).

For all three host-guest carbon dots, the absorption spectra in aqueous solutions exhibited contributions from carbon nanoparticles (more significantly in the blue/green spectral region, comparable with the absorption of carbon dots from the carbonization of PEG_900_ without the dye encapsulation, Fig. 2) and the guest dye molecules (Figs 2 and 3). However, the absorption bands of the encapsulated dyes are somewhat different from those of their corresponding free molecules, likely reflecting effects of the different environment in the hosting carbon nanoparticles. For example, the absorption of the CV in CV@CDots is much broader in comparison with that of the free dye molecules, both in aqueous solutions (Fig. 2). Similar encapsulation effects were observed in fluorescence spectra of the host-guest carbon dots. The spectra were found to be excitation wavelength dependent, as shown in Fig. 2 for example, which might be as expected considering the solid-like environment around the guest dye molecules in the hosting carbon nanoparticles (namely the molecules are each in a slightly different surrounding in a “solid-state solution”, a classical case for excitation wavelength dependent fluorescence emissions). Similarly for ZnPc@CDots in aqueous solution excited at its absorption peak, the fluorescence band is broader and red-shifted from that of the free ZnPc molecules (Fig. 3).

**Figure 4:**
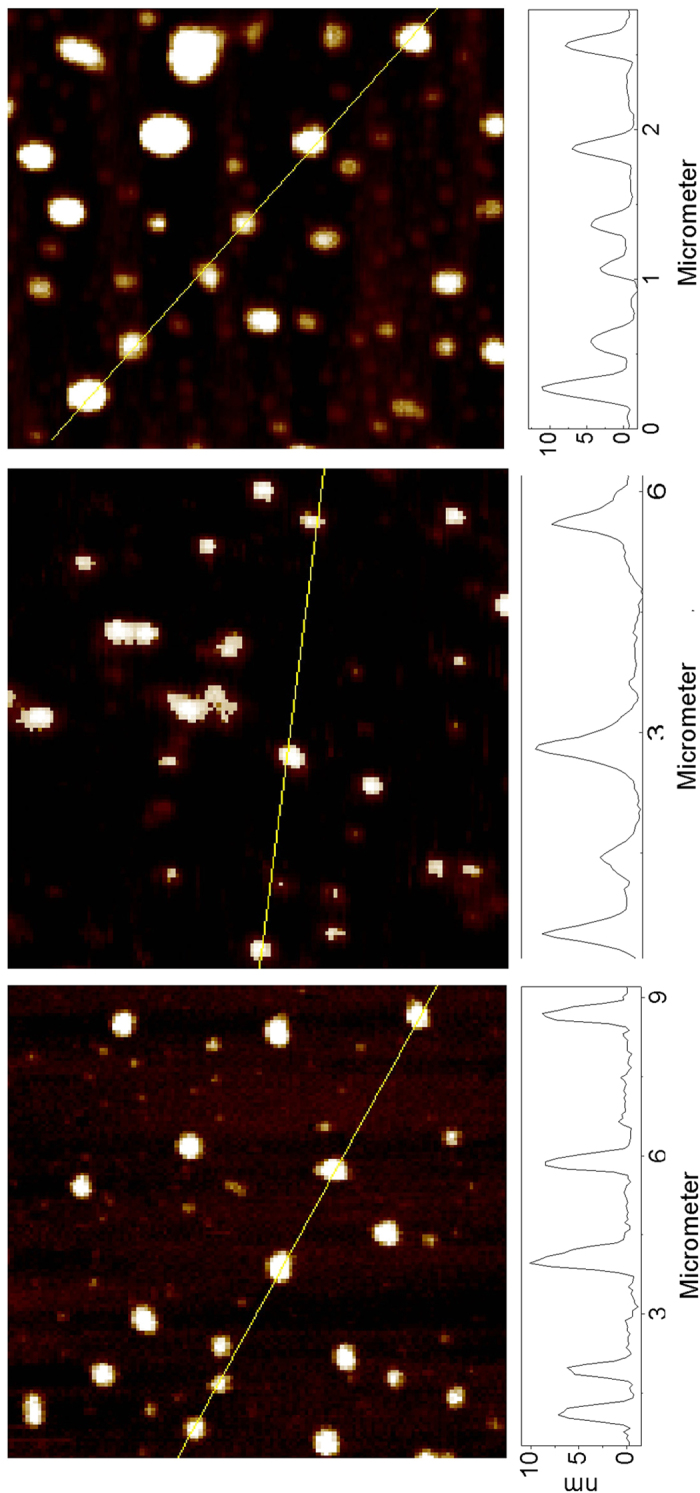
AFM images of CV@CDots (top), NB@CDots (middle), and ZnPc@CDots (bottom).

The aqueous solutions of the host-guest carbon dots were diluted for the preparation of specimens on mica substrate for atomic force microscopy (AFM) characterization. Shown in Fig. 4 are the results for CV@CDots, NB@CDots, and ZnPc@CDots. According to image height analyses, these host-guest carbon dots synthesized from thermal carbonization reactions are not as uniform in size as those from the surface chemical functionalization of pre-processed carbon nanoparticles reported previously^6^,^22^, though still relatively narrowly distributed. Most of these host-guest carbon dots are small, with their overall size profiles on the order of 10 nm or less (Fig. 4).

**Figure 5:**
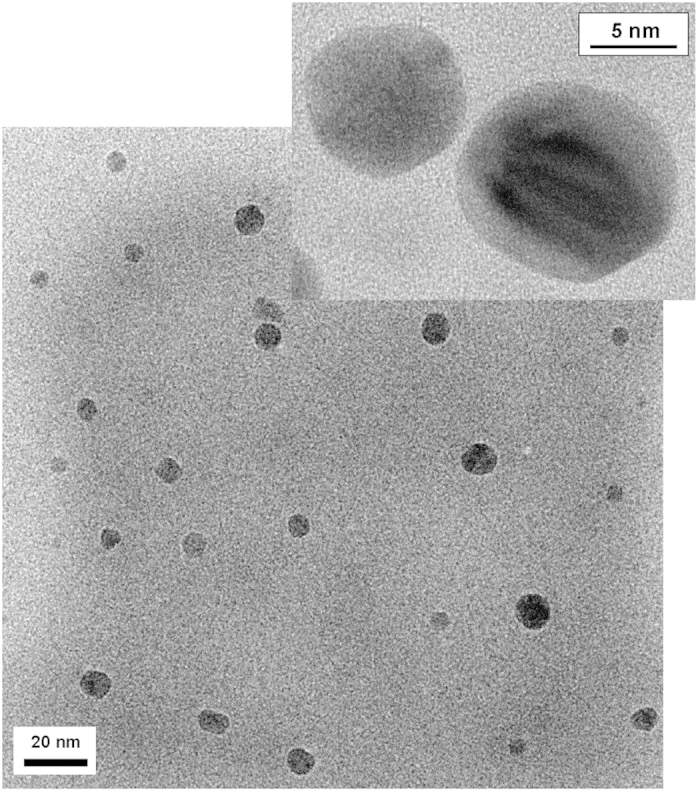
TEM images (high-resolution in the inset) of NB@CDots on silicon oxide-coated copper grid.

The carbon nanoparticle cores in the host-guest carbon dots are likely somewhat smaller than the overall dot profiles estimated from the height analysis of AFM images, as the latter may also include contributions of the organic species on carbon particle surface that survived the thermal carbonization processing. The expected significant contrast between the carbon core and surface organic species was exploited in the probing of the carbon nanoparticles by using transmission electron microscopy (TEM). For NB@CDots as an example, the TEM specimen was prepared such that a few drops of a dilute sample solution were deposited onto a silicon oxide-coated copper grid, followed by careful evaporation of the solvent. The imaging experiments were performed on a high-resolution TEM instrument (Hitachi H-9500). The results shown in Fig. 5 suggest that the NB-encapsulated carbon dots with residual PEG molecules as surface passivation moieties (confirmed by the significant PEG carbon peaks in ^13^C NMR analyses) are well-dispersed and that the carbon nanoparticle cores are size-wise small and relatively narrowly distributed.

For the host-guest carbon dots in aqueous solutions, the fluorescence quantum yields of the encapsulated dyes were evaluated against those of their free counterparts. Mechanistically, the observed fluorescence emissions from the guest dyes were due to their intrinsic electronic transition properties, not induced by the host carbon dots. However, the carbon pool environment in the hosting carbon dots could have meaningful effects on the fluorescence properties of the guest dyes. Among the three selected dyes, CV is soluble in water^23^, NB less so and only weakly fluorescent in an aqueous environment^24^, and ZnPc soluble in organic solvents^23^. Generally the results suggested that the fluorescence quantum yields of CV and NB as guests in the host-guest carbon dots were similar to those of free CV and NB molecules, respectively, all in aqueous solutions. More specifically for CV, it is known in the literature that its fluorescence quantum yields in aqueous solutions are somewhat concentration dependent, higher in a more dilute solution, yet overall about 40% lower than the yields in methanol^25^. The observed similar fluorescence quantum yields between the encapsulated and free CV molecules might be due to the opposing effects of a relatively higher CV concentration and more non-aqueous environment in CV@CDots, which decreases and enhances the quantum yields, respectively. However, for NB@CDots in an aqueous solution, the estimated fluorescence quantum yields of the guest NB were higher than that of free NB molecules in water (on the order of 0.01)^24^ but still significantly lower than that in ethanol (around 0.27)^23^, probably suggesting that the environment for the encapsulated NB is not entirely free from water. Similarly, the fluorescence quantum yields of ZnPc as guest in the host-guest carbon dots in an aqueous solution were also significantly lower than those of free ZnPc molecules in organic solvents, likely also due to the exposure of the encapsulated ZnPc to water (because the ZnPc fluorescence in a polar organic solvent is apparently quenched efficiently by the addition of water). Therefore, in further investigations the fluorescence properties of these water-sensitive dyes may be used to study the local environment in the core carbon nanoparticles in the host-guest carbon dots. Experimentally, more effort is needed to correct the light scattering effect in aqueous solutions of the host-guest carbon dots for a more accurate determination of the fluorescence quantum yields of the encapsulated dyes.

Conceptually similar to endofullerenes that have expanded the horizons of the fullerene field^13^, the host-guest carbon dots represent a new QD-like nanoarchitecture for materials properties and functions beyond those achieved with the original carbon dots. The extension of absorption and fluorescence coverage of carbon dots into the red/near-IR spectral regions with the relevant dyes as guest in this work serves as a representative example for the potential and versatile nature of the host-guest carbon dots platform. Such a new platform is expected to significantly broaden the reach of the already rapidly advancing carbon dots research and development.

## Additional Information

**How to cite this article**: Sun, Y.-P. *et al.* Host-Guest Carbon Dots for Enhanced Optical Properties and Beyond. *Sci. Rep.* **5**, 12354; doi: 10.1038/srep12354 (2015).

## Figures and Tables

**Figure 1:**
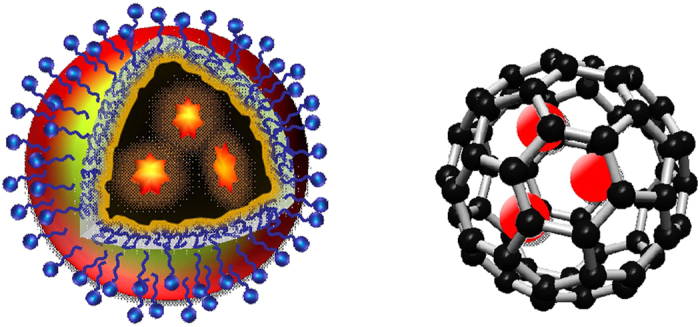
A carbon dot with encapsulated species (host-guest carbon dot, left) *verses* an endofullerene (right).
